# Impact of first-trimester ultrasound on early detection of major fetal anomalies: Nationwide population-based study of over 1 million pregnancies

**DOI:** 10.1371/journal.pmed.1004709

**Published:** 2025-11-25

**Authors:** Jehan N. Karim, Jennifer M. Broughan, Nicholas Aldridge, Pranav Pandya, Annette McHugh, Aris T. Papageorghiou, for the ACCEPTS study group

**Affiliations:** 1 Nuffield Department of Women’s & Reproductive Health, University of Oxford, Oxford, United Kingdom; 2 National Congenital Anomaly and Rare Disease Registration Service (NCARDRS), NHS England, Redditch, United Kingdom; 3 NHS Fetal Anomaly Screening Programme, NHS England, London, United Kingdom; 4 Fetal Medicine Unit, University College London Hospitals NHS Foundation Trust, London, United Kingdom; 5 Oxford Maternal and Perinatal Health Institute, Green Templeton College, University of Oxford, Oxford, United Kingdom; University of Manchester, UNITED KINGDOM OF GREAT BRITAIN AND NORTHERN IRELAND

## Abstract

**Background:**

Major fetal anomalies are an important cause of perinatal morbidity and mortality. While routine second-trimester ultrasound screening around 20 weeks is the current standard, advances in imaging have enabled earlier anatomical assessment in the first trimester. Despite increasing practice of early screening in England, there is no national policy recommending first-trimester anatomical evaluation, and little is known about its impact on detection rates at population level. Our aim was to examine if different policies of fetal anatomical ultrasound practice have an impact on earlier diagnosis of major fetal anomalies.

**Methods and findings:**

We conducted a nationwide, population-based study linking data from a national survey of first-trimester ultrasound protocols in all NHS maternity units in England with congenital anomaly registration data from the National Congenital Anomaly and Rare Disease Registration Service (NCARDRS) for pregnancies between April 2017 and March 2019. NHS trusts were classified into four protocol groups: no anatomical assessment, basic, advanced, and extended anatomical protocols. We evaluated the proportion of 14 predefined major congenital anomalies detected prior to 16 weeks’ gestation across these groups. A total of 1,030,224 pregnancies were included from 110 NHS trusts (84% response rate), with 5,895 fetuses affected by one of the anomalies of interest. First-trimester anatomical assessment was routinely conducted in 75% of trusts, though the scope varied. Overall, 32.7% (95% CI 31.5–33.9) of anomalies were detected before 16 weeks, with detection rates increasing stepwise by protocol detail: 27.7% (95% CI 25.4–30.0) in trusts with no protocol to 40.4% (95% CI 37.3–43.4) in those with extended protocols (*p* < 0.0001 for trend). Conditions such as acrania, exomphalos, and gastroschisis were commonly detected early regardless of protocol, whereas for anomalies such as spina bifida, limb reduction defects, and major cardiac malformations, detection was significantly higher in centers employing detailed first-trimester anatomical protocols. Due to data access restrictions and confidentiality considerations, analyses were conducted at the level of protocol group rather than individual hospitals. Hospital-level characteristics, including sonographer expertise and patient population risk, could not be adjusted for and may act as confounders.

**Conclusions:**

More detailed first-trimester anatomical screening protocols are associated with significantly higher early detection rates of major fetal anomalies. While current practices vary considerably across England, this study provides population-level evidence suggesting that systematic first-trimester screening could improve the timeliness of anomaly detection. These findings support the consideration of standardized national guidance to reduce inequity and enhance prenatal care.

## Author summary

**Why was this study done?:**

- Serious structural problems in the baby (congenital anomalies) affect about 2%–3% of pregnancies, and are screened for at the 20-week scan offered to all women.
- In England, most women also receive a scan at 11–14 weeks, and although there is no national recommendation to check fetal anatomy at this stage, many of the anomalies can now be detected earlier due to improved ultrasound technology.
- We wanted to find out whether hospitals that routinely assess fetal anatomy at 11–14 weeks detect more anomalies earlier than those that do not.

**What did the researchers do and find?:**

- We analyzed over 1 million pregnancies in England and compared early detection rates of 14 serious fetal anomalies across hospitals with different ultrasound policies.
- Hospitals using more detailed early ultrasound protocols detected significantly more anomalies before 16 weeks of pregnancy.
- In hospitals with the most detailed protocols, early detection was around 40%, compared to 28% in hospitals with no formal anatomical scan.
- Early detection varied by condition: some anomalies (like acrania) were commonly found early regardless of policy, while others (like heart defects or limb abnormalities) were more likely to be picked up only in hospitals using extended protocols.

**What do these findings mean?:**

- Adding a structured assessment of fetal anatomy to first-trimester ultrasound may help improve early diagnosis of serious conditions.
- A limitation of this study is that we did not have information on the scan used for each individual pregnancy, so we grouped hospitals by their reported policies, which may not fully reflect real-life variation in practice or skill.
- A national policy could help ensure all women in England have the same access to early fetal screening, reducing variation based on where they live.

## Abstract 3

In a population-based study, Jehan Karim and colleagues show that different policies of fetal anatomical ultrasound practice have an impact on the time of diagnosis of major fetal anomalies in England.

## Introduction

Approximately 2%–3% of all newborns are affected by a congenital anomaly, and this represents a significant cause of fetal, neonatal, childhood, and adult morbidity and mortality [1,2]. In most developed countries, prenatal ultrasound for fetal anatomical assessment is offered to all pregnant women around 20 weeks gestation as recommended by WHO [3]. The routine 20-week ultrasound scan aims to detect congenital anomalies [4,5], and recent data demonstrate that such screening programs detect about two-thirds of babies with a congenital anomaly before birth [6].

While second-trimester screening for anomalies at around 20 weeks represents the current gold standard practice, the majority of fetal organogenesis is completed by 10 weeks gestational age [7,8]. In addition, technological improvements in ultrasound image quality now allow earlier visualization of fetal anatomy [9,10]. This means that a significant proportion of major anomalies are detectable earlier, at 11–14 weeks of gestation, in both low- and high-risk populations [11–14].

On this basis, international guidelines have begun advocating for first-trimester anomaly screening as an addition to routine clinical practice [15,16]. Nevertheless, no current recommendations for such early anatomical screening exist in most countries, including the USA and England. This is because it is not known whether such a policy would provide benefit within the context of existing screening programmes.

In England, all women are offered a scan at 11–14 weeks for confirmation of fetal viability, measurement of fetal crown-rump length (CRL) for gestational age estimation, identification of chorionicity in multiple pregnancies, and measurement of fetal nuchal translucency (NT) thickness for women who accept the offer of a “Combined Screening Test” for Down’s, Edwards and Patau’s Syndromes [17]. Crucially, there are no national recommendations for fetal anatomical assessment at this gestation, but studies suggest that screening for fetal anomalies is taking place in many centers. There is a lack of supporting evidence on (i) the impact of such ad-hoc screening at a population-level on the timing of fetal anomaly diagnosis and (ii) on how the screening should be optimally performed.

The objectives of this study were to evaluate whether first-trimester examination of fetal anatomy has an impact on the earlier diagnosis of major fetal anomalies. This was examined by establishing whether differences in first-trimester examination protocols are associated with differences in the proportion of major fetal anomalies identified early in pregnancy.

## Methods

This study was conducted in two parts. The first involved undertaking a nationwide survey of current first-trimester ultrasound practice in all NHS maternity units in England in order to establish current first-trimester examination protocols. These findings were then linked with data from the National Congenital Anomaly and Rare Disease Registration Service (NCARDRS) database, to determine the impact of different first-trimester screening policies on the timing of diagnosis for a priori selected major fetal congenital anomalies. This study is reported as per the Reporting of Studies Conducted using Observational Routinely-Collected Data (RECORD) guideline (S1 RECORD Checklist).

### Nationwide survey development, validation, and piloting

In collaboration with the Fetal Anomaly Screening Program (FASP), a part of Public Health England (and now a part of NHS England), we designed a national survey to assess current first-trimester ultrasound practices. The survey included 36 questions across four domains: (1) current first-trimester ultrasound protocols; (2) local policies; (3) inclusion of an early evaluation of fetal anatomy; and (3) availability of resources (S1 Appendix). The format and question style of the questionnaire mirrored previous national surveys of antenatal ultrasound practice commissioned by the UK National Screening Committee [18,19]. This continuity in format was intended to provide familiarity for the staff completing the questionnaire, ensuring consistency and ease of completion. The survey underwent a multi-stage validation and piloting process to ensure its reliability and validity:

1. Document review by core members of the research team to ensure clarity and relevance of the questions.
2. External review and piloting by a group of research midwives, sonographers, and fetal medicine specialists with academic knowledge of the topic. This group, external to the research team, assessed the questionnaire for face validity (relevance, formatting, readability, clarity, and appropriateness of the questionnaire) and content validity (extent to which the survey covered all relevant content areas).
3. To test reproducibility (test–retest reliability), the same individuals as in (2) completed the survey multiple times. Their responses were analyzed for inter-rater reliability (consistency within and between respondents) and internal consistency (coherence of the survey’s content).
4. The survey was further piloted and validated by JK working with sonographers with an interest in antenatal ultrasound at the British Medical Ultrasound Society’s annual conference (Manchester, November 2018; *n* = 17). Participants provided constructive feedback, and responses analyzed to ensure understanding of the questions, and consistency with expected results.
5. Final document review and approval by members of FASP (led by AM, PP).

The finalized survey was distributed electronically to all NHS maternity trusts in England (*n* = 131) on January 25th, 2019. To maximize response rates an electronic reminder was sent to all trusts by FASP (February 2019); and the study team contacted individual trusts with outstanding responses (April 2019). In cases where first-trimester ultrasound examination protocols were different amongst units forming part of one trust, we requested a separate questionnaire for each ultrasound department. Survey submission closed in June 2019.

Data were anonymously analyzed using descriptive statistics. Responses from trusts in the nine different regions (as defined by Public Health England) were compared using Chi-squared tests. Additionally, responses received from different levels of care (tertiary, secondary, community) were compared using descriptive statistics. For the purposes of this analysis, we focus on first-trimester examination protocols implemented by each trust.

### Classification of NHS ultrasound units based on first-trimester screening protocol

To examine the impact of different first-trimester anomaly screening policies, we aggregated data from NHS Hospital trusts that responded to the nationwide survey. These trusts were categorized into four groups based on the type of examination protocol in use:

Group A: no formal examination of the fetal anatomy during the first-trimester ultrasound scan
Group B: basic anatomical assessment, defined as a formal examination of at least one of the following structures: the fetal head, the fetal limbs, and/or cord insertion into the fetal abdomen.
Group C: advanced anatomical protocol, defined as the “basic” protocol with additional evaluation of the fetal stomach or bladder or both.
Group D: extended anatomical protocol defined as the “advanced” protocol with the addition of an examination of the fetal heart, fetal spine, and/or the fetal face. This group included units with the most detailed first-trimester anatomical examination protocols in England.

The NCARDRS team was provided with the list of NHS units classified into each first-trimester protocol group (A, B, C, D) but blinded to the meaning of each group.

### NCARDRS congenital anomaly registration data

The survey data above were linked to data regarding the number of babies with a diagnosis of a congenital anomaly and timing of that diagnosis from the NCARDRS. NCARDRS, part of the National Disease Registration Service in England, collects data on congenital anomalies and rare diseases [20] and performs national audits to support the NHS FASP [21]. NCARDRS registers and monitors epidemiological information on the prevalence, timing of diagnosis, and outcome of babies and fetuses with a diagnosed congenital anomaly, including both antenatal and postnatal diagnoses. Reporting of this information became a mandatory requirement for all NHS hospital trusts in England in 2017. Based on the clinical information provided by individual hospital trusts, NCARDRS codes anomalies using WHO’s International Classification of Diseases 10th revision (ICD-10) with the British Paediatric Association Adaptation, which gives supplementary one-digit extensions to ICD-10 codes to allow greater specificity of coding [20]. For the purposes of this study, data regarding pregnancies affected by one of the following congenital anomalies of interest were extracted from the NCARDRS database using relevant ICD-10 codes (either individual codes or aggregates of multiple codes, S2 Appendix): acrania/anencephaly/exencephaly/iniencephaly, encephalocele, exomphalos (omphalocele), gastroschisis, spina bifida, facial clefts (cleft lip and/or palate), congenital diaphragmatic hernia, bilateral renal agenesis, lethal skeletal dysplasias, limb reduction defects, hypoplastic left heart syndrome, atrio-ventricular septal defect, tetralogy of Fallot and transposition of great arteries. This list was compiled as a combination of the 12 structural anomalies which are the focus of the current FASP second-trimester anomaly screening programme and an additional two anomalies (encephalocele and limb reduction defects), which are considered to be of particular interest to the early anatomy scan.

Details of fetuses with one of these congenital anomalies and with an expected date of delivery between April 2017 and March 2019 (inclusive) were aggregated by anomaly and scan protocol group (A–D). The gestational age at which the diagnosis was first suspected or diagnosed was identified as either: (i) prior to 16 weeks (this covers the period of routine screening at 11–14 weeks in addition to referral), (ii) between 16 and 23 + 6 weeks, (iii) after 24 weeks but prior to birth. Babies with congenital anomalies identified after birth were not included as part of the analysis, as the objective of this study was to examine the impact of first-trimester ultrasound examination on the timing of antenatally detected anomalies.

We excluded from the analysis fetuses where the gestational age of diagnosis was unknown, fetal losses prior to screening, and those that did not book in time for screening (greater than 112 days gestational age at time of booking), as they would not have been offered a first-trimester ultrasound scan as part of their antenatal care. The data provided was restricted to those NHS hospitals that responded to the nationwide survey of practice (84% of all trusts), as details regarding first-trimester anomaly screening protocols were only available for these hospitals.

Individual hospital-level analysis was not undertaken due to the low prevalence of certain anomalies, which could risk identification of individual patients.

First, the rate of each anomaly was calculated. The antenatal detection rate prior to 16 weeks and after 16 weeks (until birth) was calculated for the combined group of 14 anomalies (composite) and for the selected individual anomaly for all included NHS hospitals, irrespective of the first-trimester protocol in use. This analysis was then repeated for each protocol group, respectively (A, B, C, D). Early detection rates were compared using a Chi-squared test for the composite and for fetal anomaly groups individually. Significance was set at *p* < 0.05. All statistical analysis was performed using StatsDirect statistical software version 3.3.0. (StatsDirect Ltd, Altrincham, UK). A prospective analysis plan was developed and submitted prior to data analysis and is available in S1 Protocol.

### Ethical approval

Ethics approval was obtained after a full review by the North West—Preston Research Ethics Committee (21/NW/0173) in March 2021. Additionally, approval for the conduct of this research was formally granted by the UK National Disease Registration Service Review Panel on behalf of Public Health England. Members of the immediate research group (JK and AP) were granted honorary academic contracts with NCARDRS to facilitate this partnership.

### Patient and public involvement statement

The work described was developed and undertaken as part of the ACCEPTS group (NIHR HTA grant 17/19/10), which included involvement from four UK-based patient advocacy groups including Antenatal Results and Choices, the National Childbirth Trust, SHINE, and Tiny Tickers. This study was conducted alongside a national, prospective, survey conducted at 10 NHS trusts within the UK, assessing the attitudes of parents towards the introduction of a first-trimester anomaly scan to routine care. There were no patients consulted directly regarding the development or undertaking of this study.

## Results

Of the 131 NHS hospital trusts participating in the NHS England antenatal screening programme, responses were received from 110 NHS trusts (response rate 84%). Completed questionnaires were submitted by more than one ultrasound unit within the same NHS trust in 8 cases, meaning 118 responses were received. In five cases, the first-trimester protocols in the two units submitting responses from the same trust were identical; in three cases the individual protocols within the same trust were different, and for the purposes of this analysis, the most detailed of the two protocols was considered. Thus, analysis from 110 NHS hospital trusts was undertaken, and based on first-trimester examination protocols, these were classified into Group A (*n* = 27), Group B (*n* = 22), Group C (*n* = 45), and Group D (*n* = 16). The responses obtained reflected a variety of healthcare settings, including district general hospitals (*n* = 74, 67%), university-affiliated and academic centers (*n* = 35, 32%), and tertiary care units (*n* = 12, 11%), and represented trusts from all nine designated health regions in England (Table 1).

**Table 1:** Response rates of trusts by regions as defined by Public Health England (PHE) at time of study.

Region	Number of responding trusts/Total number of NHS trusts in region (*n*/*n*, %)
North East England	4/9 (44)
North West England	14/19 (74)
Yorkshire and Humber	13/13 (100)
East Midlands	11/13 (85)
West Midlands	11/13 (85)
East of England	8/11 (73)
London	17/18 (94)
South East England	16/18 (89)
South West England	16/17 (94)

Despite the absence of a national recommendation, 83 of the 110 responding trusts (75%) routinely perform some form of first-trimester anatomy assessment, and this takes place across all types of units (100% of tertiary care centers, 80% of university and academic centers, and 69% of district general hospitals). Amongst the 83 centers performing anatomical assessment, there were variations in what anatomy was routinely assessed: fetal head (99% of reported protocols), limbs (95%), cord insertion (91%), stomach (72%), bladder (65%), heart (17%), spine (16%), thorax (14%) neck (12%), face (7%), and kidneys (6%, S3 Appendix). Of the 110 trusts responding, 94% primarily use *trans-*abdominal ultrasound for screening with the offer of a *trans-*vaginal scan when required for improved visualization, while the remainder offer exclusively *trans-*abdominal imaging. The majority of units (97%) allocate 20–30 min for their first-trimester ultrasound scan appointment, regardless of whether a formal assessment of fetal anatomy is conducted and how such an examination is performed.

Using the NCARDRS database, we identified that 1,030,224 pregnancies had pregnancy care between April 2017 to March 2019 (inclusive) in the 110 NHS trusts included in the study.

Overall, 6,808 fetuses were identified as having a congenital anomaly of interest, of which 913 (13.4%) were excluded from the analysis because either the gestational age of diagnosis was unknown, a fetal loss took place prior to screening, or the pregnancy was not booked in time for first-trimester screening (Group A: *n* = 206/1720 (12.0%), Group B: *n* = 155/1247 (12.4%), Group C: *n* = 374/2604 (14.4%), Group D: *n* = 178/1237 (14.4%)). As a result, 5,895 fetuses were identified as being affected by one of the 14 prespecified anomalies antenatally and were therefore included in the analysis. The prevalence of each of the anomalies was consistent with that expected based on available data published data from population-wide anomaly databases, NCARDRS [6] and EUROCAT [22].

Of the 5,895 fetal anomalies identified, 1,929 (32.7%, 95% Confidence Interval (95% CI) 31.5–33.9) were suspected or diagnosed prior to 16 weeks gestational age. Early detection rates for the 14 anomalies (composite) was lowest in Group A (27.7%, 95% CI 25.4–30.0), with a stepwise increase in detection in Group B (31.2%, 95% CI 28.5–34.1), Group C (33.2%, 95% CI 31.3–35.2) and Group D (40.4%, 95% CI 37.3–43.4, Chi-squared for trend *P* < 0.0001, Table 2).

**Table 2:** Early detection of 14 fetal congenital anomalies based on the type of first-trimester anatomical screening policy.

	First-trimester screening protocol group				
Parameter	Group A No protocol	Group B Basic protocol	Group C Advanced protocol	Group D Extended protocol	All
NHS units included (*n*)	27	22	45	16	110
Total number of anomalies (*n*)	1,514	1,092	2,230	1,059	5,895
Anomalies detected prior to 16 weeks (*n*)	419	341	741	428	1929
Detection rate prior to 16 weeks (%, 95% CI)	27.7 (25.4–30.0)	31.2 (28.5–34.1)	33.2 (31.3–35.2)	40.4 (37.3–43.4)	32.7 (31.5–33.9)

Chi-squared test for linear trend demonstrates increases in sensitivity with increasing detail of protocol (*p* < 0.001).

Secondary analysis broadly identified three clusters: (i) anomalies where the majority of affected fetuses are currently identified in the first trimester regardless of screening policy, (ii) anomalies where detection was higher in units routinely using a first-trimester anatomical examination protocol, and (iii) anomalies which are poorly detected in the first trimester, regardless of policy (Table 3).

**Table 3:** Prevalence and detection rates of prespecified fetal anomalies <16 weeks gestational age according to first-trimester anatomy screening policy.

Anomaly	Prevalence (per 10,000 births)^A^	Total number of anomalies^B^ (*n*)	Anomalies detected prior to 16 weeks by units using NO protocol (%)	Anomalies detected prior to 16 weeks by units using a protocol (%)	*P*-value^C^
**Fetal anomalies where detection rates <16 weeks are high regardless of screening policy:**					
Acrania	5.9	572	95.0	94.4	0.6
Exomphalos	7.0	653	89.4	86.9	0.5
Gastroschisis	3.3	312	75.9	83.0	0.2
**Fetal anomalies where detection rates <16 weeks associated with screening policy:**					
Encephalocele	1.8	108	25.0	67.9	*p* < 0.001
Facial clefts^*^	10.8	982	2.9	18.3^*^	*p* < 0.001
Spina bifida^*^	6.6	607	12.6	30.4^*^	*p* < 0.001
Limb reduction anomalies	3.6	193	8.8	43.8	0.003
AVSD^*^	7.7	654	10.2	42.1^*^	*p* < 0.001
HLHS^*^	3.5	310	4.1	31.8^*^	*p* < 0.001
TOF^*^	5.3	479	2.2	20.0^*^	*p* < 0.001
Transposition of great arteries^*^	4.1	381	0.9	7.3^*^	0.01
**Fetal anomalies where detection rates <16 weeks are low regardless of screening policy:**					
Lethal skeletal dysplasias	1.7	147	22.0	21.8	0.6
Congenital diaphragmatic hernia	3.9	365	7.8	13.2	0.4
Bilateral renal agenesis	1.5	132	11.6	12.7	0.9

^A^Prevalence was calculated as number of anomalies relative to the number of deliveries in the study population over the time period.

^B^Total number of anomalies includes anomalies eligible for detection and excludes pregnancies where gestational age of diagnosis was unknown, fetal losses prior to screening, and those that did not book in time for screening.

^C^*P*-values resulting from pair-wise comparison of centers evaluating first-trimester anatomy using a protocol (groups B + C + D) with those who do not use one (Group A) based on Chi-squared test.

*For these anomalies, pair-wise comparison was made between those centers evaluating the fetal heart, face, and spine (Group D) against those units which do not routinely evaluate these structures (Groups A, B, and C). AVSD, atrioventricular septal defect; HLHS, hypoplastic left heart syndrome; TOF, tetralogy of Fallot.

## Discussion

In this study, we have demonstrated that screening for fetal anomalies in the first trimester impacts the gestational age at which anomalies are diagnosed within a population-based screening program. We present a comprehensive analysis of first-trimester ultrasound practices and how they relate to the timing of antenatal detection of fetal congenital anomalies. By examining population-based national data from 110 NHS hospitals in England, and linking this to the national congenital anomalies database, we have established that earlier anatomical screening results in higher detection rates of fetal anomalies in early pregnancy (prior to 16 weeks gestation) at the population-level.

In addition, we have identified significant inequity in healthcare provision: three-quarters of hospitals routinely offer women first-trimester assessment of fetal anatomy despite no national guidance to that effect. It appears that this absence of national recommendations has led to individual hospitals establishing screening policies independently, so that there is considerable variation in practice. While it is clear that units have been able to establish this practice within existing resources, the differences in the standard of antenatal screening have resulted in significant regional and local inequities in maternal care, with differential fetal anomaly detection rates prior to 16 weeks gestation.

Current screening guidance recommends assessment of fetal viability, measurement of the fetal CRL, and measurement of fetal NT [17]. However, as ultrasound imaging has increased in resolution, anatomical pathologies are often instantly visible to the screening sonographer. The finding that a significant majority of units currently undertake a first-trimester anatomical screening scan, despite no such requirement, is striking. This suggests that the technological advances of ultrasound and sonographer training are outpacing policies and recommendations. The resulting variation in practice range from no formal assessment to extensive and detailed fetal examination.

Differences in screening policies revealed by our survey were also reflected in prescreening information given, how inconclusive scans are followed up, and what referral pathways exist. Hospitals more likely to evaluate fetal anatomy were also more likely to have formal policies for performing anatomical assessment. As an example, in region A, all responding trusts performed early anatomy screening and almost 90% had a formal protocol; while in region I, less than half of the responding units offered routine anatomical screening and a formal policy was present in less than one-third. These findings strongly suggest the emergence of “ad-hoc” screening, highlighting important inequities in antenatal options offered to women based on where they live. In our view, consideration of a national first-trimester anatomical screening standard would be expected to reduce some of this variation and promote equity.

Our analysis focused *a priori* on the detection of 14 major congenital anomalies; 12 of these are currently the focus of the second-trimester FASP. We found that approximately one third (32.7%) of these anomalies were detected prior to 16 weeks gestational age in England. This is an important finding, as most patients and healthcare providers consider that assessment for congenital fetal anomalies takes place in the second trimester. The fact that about one-third of these major anomalies are diagnosed at earlier gestations has important implications for informed prescreening consent, parental expectations of possible outcomes after first-trimester ultrasound, and provision of referral pathways. While the highest detection rates were seen in those centers performing detailed first-trimester ultrasound using formalized policies and protocols (40.4%, Group D), a significant proportion of fetal anomalies are diagnosed essentially incidentally, i.e., without formal anatomical assessment (27.7%, Group A).

There is evidence to suggest that the great majority of parents favor access to earlier screening, as it provides early reassurance or diagnosis [23–25]. For those parents with an affected pregnancy, an early diagnosis offers advantages such as additional time for genetic testing, multidisciplinary input, discussions around in-utero therapy options, and informed and balanced decision-making around pregnancy management. For those women considering a termination of pregnancy, having this performed at earlier gestations may be safer with a significantly reduced risk of maternal complications and length of hospital stay [26–30].

Previous reviews assessing first-trimester anatomical screening have suggested that conditions can be grouped into those nearly always detectable, those that are potentially detectable depending on maternal, fetal, sonographer, and equipment factors; and those that are rarely identifiable [11,31]. Our findings concur with this suggested model using real-world data: for example, over 80% of fetuses affected by acrania, exomphalos, and gastroschisis are identified prior to 16 weeks gestational age suggesting that the fetal skull and fetal cord insertion are examined even when anomaly screening is not a formal objective for the scan. Notably, these structures are readily evident in the standard mid-sagittal view required for measurement of the fetal CRL, meaning these conditions are often visualized incidentally, even when formal assessment is not intended. We identified a further group of eight conditions where a formal anatomical protocol was associated with improvements in detection rates, consistent with findings from reviews of smaller studies [14,31,32]. Finally, detection rates for some anomalies before 16 weeks gestation were poor regardless of the imaging protocol used, namely bilateral renal agenesis, congenital diaphragmatic hernia, and lethal skeletal dysplasias. Here, the small fetal size, evolution through pregnancy, and impact on amniotic fluid volumes resulted in later detection [7,8].

This study is the first to characterize first-trimester anomaly screening practice at population level, and to examine the impact of screening method on fetal anomaly diagnosis. The main strengths are the high rate of population-level coverage, with over 80% of NHS Hospital trusts in England, including over 1 million pregnancies and in every region. Although a limitation, the non-response rate was very low; it is possible that non-responders had a lower interest in first-trimester anatomical screening; or unable to support first-trimester services in their current form. Even assuming that none of the non-responders undertake any form of first-trimester screening for fetal anatomy, this would still result in 64% of trusts performing such screening, and the associations are unlikely to be changed. The use of a national anomaly registry with independent and anonymized data collection is another strength, as all NHS trusts in England are mandated to report, ensuring that data were as complete as possible. Limitations of the analysis include that we did not undertake hospital-level analysis, instead grouping into four clusters (A, B, C, D) based on the first-trimester anatomical examination protocol. This was a requirement of our review board, as the rarity of individual abnormalities could inadvertently lead to the identification of an individual in a small unit. This grouping has some limitations, as use of a screening policy alone is just one indicator of quality; other factors that may influence screening were not taken into account, such as the time given for examinations (similar between units); or skill of individual sonographers in each unit (not possible to measure at this large scale); or modernity of ultrasound equipment used for screening. In addition, protocols in each group were broadly similar rather than identical.

The data from the Nationwide Survey of Practice were collected in early 2019, before the COVID-19 pandemic, and therefore reflect screening practices at that time. Data analyzed from NCARDRS were from April 2017 (after the reporting became nationally mandated) to March 2019 (inclusive), and we assumed broad consistency in practice over this 2-year time period in all trusts; we believe this is reasonable given no new national guidance was released over this time.

These limitations would be more critical if the aim of this study was to determine with accuracy the sensitivity of the first-trimester scan in England. Rather, the objective was to explore whether reported changes in practice towards early anomaly screening reflected how and when fetal congenital anomalies are diagnosed. The results suggest that this is in fact the case: despite an absence of formal recommendations, a programme of first-trimester anatomical assessment is already underway in most centers in England. This ad hoc approach to screening may be lacking consistency and standardization between trusts, but is having a real-world impact on the diagnosis of fetal congenital anomalies in pregnant women.

Finally, an important limitation is that the NCARDRS database captures only those fetuses affected by congenital anomalies. This has supported an accurate assessment of first-trimester antenatal ultrasound screening sensitivity. However, without data on pregnancies impacted by a false positive result, assessment of specificity could not be undertaken. Based on the available literature and recent large-scale systematic reviews and meta-analyses, estimates suggest that the false positive rate for first-trimester anomaly screening is low, with estimates of 2.1% and 3.1% for major cardiac anomalies and noncardiac anomalies, respectively [31,32]. The consequences of a false positive screening result for parents will include referral to a fetal medicine specialist for ultrasound assessment (now as a diagnostic test) which may refute the original screening result or lead to additional serial ultrasound assessments, and possible offer of genetic testing, all of which may cause unnecessary anxiety and uncertainty for parents as well as additional healthcare cost. In the first trimester, there is additional complexity, as it can be difficult to identify what constitutes a false positive result as anomalies evolve. For example, it is widely accepted that the majority of bowel-only exomphalos in euploid pregnancies diagnosed in the first trimester will have resolved at later gestational ages without long-term associated complications. For these reasons, healthcare providers must be prepared to undertake appropriate counseling based on screening findings and parents well informed of the possible outcomes. A consistent nationwide approach supported by accessible patient information highlighting risks and benefits of screening, evidence-based guidelines, and educational teaching modules for healthcare providers would be expected to support informed patient consent and ensure provision of a high-quality and equitable screening service.

These nationwide data reveal significant variations in first-trimester ultrasound practices and demonstrate that more detailed screening protocols are associated with higher early detection rates of fetal anomalies. This finding highlights the need for standardized national guidelines to ensure equitable and effective antenatal care for all pregnant women. Standardizing first-trimester screening protocols within a formal extension of scope of the current screening programme could enhance early detection, improve parental counseling, and optimize pregnancy management, ultimately leading to better outcomes.

## Supporting information

S1 Protocol Statistical analysis plan that was submitted as part of the approval process. (PDF)

S1 Acknowledgements Members of the Assessing Clinical and Cost Effectiveness of Prenatal first-Trimester anomaly Screening (ACCEPTS) study group. (PDF)

S1 Appendix Nationwide Survey of First-Trimester Ultrasound Practice. (PDF)

S2 Appendix ICD-10 codes used to identify fetuses within NCARDRS database affected by one of 14 congenital anomalies of interest in this study. (PDF)

S3 Appendix Screening and protocol use in each geographical region. (PDF)

S1 RECORD Checklist RECORD statement checklist. (DOCX)

## Abbreviations

CRL: crown-rump length
FASP: Fetal Anomaly Screening Program
ICD-10: International Classification of Diseases 10th revision
NT: nuchal translucency
NCARDRS: National Congenital Anomaly and Rare Disease Registration Service
RECORD: Reporting of Studies Conducted using Observational Routinely-Collected Data
