# Supplementary material for: Impact of first-trimester ultrasound on early detection of major fetal anomalies: Nationwide population-based study of over 1 million pregnancies
Source: PLoS Med. 2025 Nov 25;22(11):e1004709. doi: 10.1371/journal.pmed.1004709 (PMC12646414; doi:10.1371/journal.pmed.1004709)
Supplement: S1 Protocol — (PDF) [file pmed.1004709.s001.pdf]

Not applicable

9.8. Sample Handling
Not applicable.

9.9. Early Discontinuation/Withdrawal of Participants
This study is not a prospective study. Instead, routinely collected data collected by NCARDRS is being used for this research study. The data being shared is de-personalised therefore withdrawal is not an issue.

9.10. Definition of End of Study
As prospective data is not being collected for this study, the end of the study will be once the objectives have been reached which we anticipate will be in January 2022.

10. SAFETY REPORTING
Not applicable for this study.

11. STATISTICS AND ANALYSIS

For the primary study objective, we will calculate the prevalence of each anomaly according to whether a first trimester protocol was used or not (Groups A vs. B+C+D). We will then determine the proportion of fetal anomalies detected prior to 16 weeks (this covers the period of routine screening at 11-14 in addition to referral), 16-24 weeks (which covers the existing second trimester anomaly scan), above 24 weeks and postnatally.

We will assess whether the existence of a protocol is associated with differences in the proportion of women receiving a diagnosis in the gestational age groups described (Chi-Squared Test for proportions). We will also assess gestational age as a continuous variable and analyze the median gestational age at diagnosis between the two groups (Mann- Whitney Test).

Sub-analysis between the three first trimester protocol types (Groups B, C, D) identified by our national survey of NHS practice will be undertaken only if significant differences between non-protocol (Group A) and protocol (Group B, C, D) are found. If possible, a sensitivity analysis will be undertaken for the primary study objective, which focuses only on NHS trusts who are considered to have provided a complete data-set report to NCARDRS.

Descriptive statistics of proportions of anomalies detected at the different gestational age windows will be reported according to the nine PHE groups, but no comparative statistical analysis will be undertaken as this would be of limited value.

Secondary objectives:

© Copyright: The University of Oxford and Oxford University Hospitals NHS Foundation Trust 2019

Firstly, we will explore the systems factors which are associated with differences in early fetal anomaly detection and these will include: the anatomical protocol used (as above), the existence of follow-up protocols for first trimester detection, time allocation to each screening assessment and additional training provided to sonographers. These will be assessed using a univariate analysis, with multi-variate analysis considered if appropriate.

Secondly, using a similar statistical approach as with the primary objective, we will determine whether performance of first trimester fetal anomaly screening impacts the timing of diagnostic testing (including amniocentesis and chorionic villus sampling) and the gestational age at which termination of pregnancy is undertaken in the subgroup of patients who opt for this management.

If feasible, a sensitivity analysis will be undertaken, which focuses only on NHS trusts that are considered to have provided a complete data-set report to NCARDRS.

11.1. Sample Size Determination

The primary objective of the study is to determine whether NHS trusts in England undertaking a protocol-based first trimester fetal anomaly scan are able to provide earlier diagnosis of fetal congenital anomalies compared to those trusts offering the current standard of care. The main statistical goal is that the sample size should be large enough to be able to demonstrate a statistical difference should this exist. Although statistical considerations are important in this regard, the availability of data through NCARDRS is critical as well. Thus, for example, relevant data only became available in 2017 (when reporting became mandatory). Based on the anticipated sample and detection rates available from our previous systematic review (Karim 2017), the sample size was estimated as follows:

The overall detection rate for major congenital anomalies when utilizing a first trimester protocol is 52%, while it was 25% when no protocol was followed. Accepting an alpha risk of 0.05 and a beta risk of 0.2 in a two-sided test, 58 subjects would be necessary in each arm (protocol vs. no protocol) for each of the anomalies. Given the estimated prevalence of the major anomalies under examination and the birth rate in the UK, we expect between the lowest number to be 60 affected patients in each arm (encephalocele). Given that the prevalence for other anomalies is higher, the numbers in each arm will exceed this, with a maximum of 400 in each arm (acrania/anencephaly). The chosen sample size is larger than most previous studies, even if each fetal anomaly is considered separately; this is in part due to a number of variables which remain uncertain such as the balance between the two groups, regional variations, and population differences. Nevertheless, the selected sample size should ensure that the risk of an alpha error is very low, and would also allow ascertainment of the secondary objectives as described.

Please also note, that the large number of individual abnormalities (minimum group size of approx. 60) will ensure patient anonymity is maintained.

12. DATA MANAGEMENT

PHE are responsible for the data. The Oxford team will be given access to the data through PHE computers and infrastructure as the data will be stored on PHE servers. None of the row-level data will be stored on University of Oxford devices. The team will only have access to the row-level data for the

© Copyright: The University of Oxford and Oxford University Hospitals NHS Foundation Trust 2019

duration of the study, once the work has been completed, the Oxford team will return the PHE laptops and will no longer have access to the raw data.

12.1. Source Data

The NCARDRS database hosts routinely collected information on fetal abnormalities and the outcomes. We are requesting access to a sub-set of this data. We do not have access to the source documents, hospital records or individual's information.

12.2. Access to Data

Direct access will be granted to authorised representatives from the Sponsor and host institution for an audit of the study to ensure compliance with regulations.

12.3. Data Recording and Record Keeping

The data provided to us will be anonymised and will be coded according to PHE policy. We do not have access to any patient information. The data provided for the analysis will be kept on secure Public Health England servers which the team will access through PHE laptops and infrastructure. At the end of the study the team will return the laptops and their access to PHE servers will be revoked.

13. QUALITY ASSURANCE PROCEDURES

The study may be monitored, or audited in accordance with the current approved protocol, GCP, relevant regulations and standard operating procedures.

13.1. 13.1 Assessment and management of risk

This research proposal is based on retrospective data collection from already established medical records. There will be no direct patient contact. The retrospective design means that the conduct of this study cannot alter the treatment they have already received. Thus, there are no direct risks nor burdens to their health. The risk to patients from this study is associated with breach of confidentiality and any harm this could cause.

13.2. Study monitoring

This study does not need to be monitored. The study involves the team accessing the de-personalised aggregated data on PHE computers and infrastructure with no participant involvement therefore monitoring should not be necessary.

13.3. Study Committees

Not applicable. This is not appropriate for a study of this size.

14. PROTOCOL DEVIATIONS

A study related deviation is a departure from the ethically approved study protocol or other study document or process (e.g. consent process or administration of study intervention) or from Good Clinical

© Copyright: The University of Oxford and Oxford University Hospitals NHS Foundation Trust 2019
