# Supplementary material for: Impact of first-trimester ultrasound on early detection of major fetal anomalies: Nationwide population-based study of over 1 million pregnancies
Source: PLoS Med. 2025 Nov 25;22(11):e1004709. doi: 10.1371/journal.pmed.1004709 (PMC12646414; doi:10.1371/journal.pmed.1004709)
Supplement: S1 Acknowledgements — (PDF) [file pmed.1004709.s002.pdf]

Members of the Assessing Clinical and Cost Effectiveness of Prenatal first Trimester anomaly Screening (ACCEPTS) study group

Clinical and study design group:
Aris T Papageorghiou (PI, University of Oxford, UK), Zarko Alfirevic (Liverpool Women's NHS Foundation Trust, UK), Trish Chudleigh (Cambridge University Hospitals NHS Foundation Trust, UK), Hilary Goodman (Hampshire Hospital NHS Foundation Trust, UK), Christos Ioannou (Oxford University Hospitals NHS Trust, UK), Heather Longworth (Liverpool Women's NHS Foundation Trust, UK), Jehan Karim (University of Oxford, UK), Kypros Nicolaides (King's College Hospital NHS Foundation Trust, UK), Pranav Pandya (University College London Hospitals NHS Foundation Trust, UK), Gordon Smith (University of Cambridge, UK), Basky Thilaganathan (St George's University Hospitals NHS Foundation Trust, UK) and Jim Thornton (University of Nottingham, UK).

Health economics group: Oliver Rivero-Arias (Lead, University of Oxford, UK), Helen Campbell (University of Oxford, UK), Ed Juszczak (University of Oxford, UK), Louise Linsell (University of Oxford, UK) and Ed Wilson (University of Cambridge, UK).

Qualitative research: Lisa Hinton (University of Cambridge, UK)

Patient Voice: Jane Fisher (Lead, Antenatal Results and Choices, UK), Elizabeth Duff (National Childbirth Trust, UK), Anne Rhodes (Tiny Tickers, UK), Gil Yaz (SHINE UK).
