# Supplementary material for: Impact of first-trimester ultrasound on early detection of major fetal anomalies: Nationwide population-based study of over 1 million pregnancies
Source: PLoS Med. 2025 Nov 25;22(11):e1004709. doi: 10.1371/journal.pmed.1004709 (PMC12646414; doi:10.1371/journal.pmed.1004709)
Supplement: S1 Appendix — (PDF) [file pmed.1004709.s003.pdf]

Appendix 1 – Nationwide Survey of First Trimester Ultrasound Practice

Dear Colleagues,

We need your help!

We are currently working on an NIHR HTA grant exploring whether first trimester fetal anomaly screening would be clinically and cost-effective for women in England.

As part of our remit, we are planning a nationwide survey of all providers of NHS maternity care. The aim is to establish an understanding of how the current first trimester scan is undertaken and determine to what extent this differs from one trust to another.

We are working with the NHS fetal anomaly screening programme (NHS FASP) in developing and distributing this questionnaire. The findings will also be shared with FASP.

We need your input at this stage of the process.

Please answer all questions:

- The questionnaire should be completed by one person for each maternity care provider.

- If you have multiple sites offering first trimester ultrasound please answer and submit one questionnaire covering both the main and satellite units if the policies are the same at each site.

- Where policies differ on separate sites, please complete separate questionnaires for each site but indicate to which main provider the satellite unit is accountable.

- The questionnaire should be completed by the unit screening support sonographer or another nominated sonographer and submitted electronically to the following email: **jehan.karim@wrh.ox.ac.uk**.

- If preferable, you may print out the form and send via post to: Dr. Jehan Karim, Level 3 Women's Centre, University of Oxford, John Radcliffe Hospital, Oxford OX3 9DU. Please send us an email to inform us that you will be sending a hard copy.

If you have any queries regarding completion of the questionnaire please contact: jehan.karim@wrh.ox.ac.uk

Thank you in advance for your support.

Jehan Karim & Aris Papageorghiou
Department of Women's and Reproductive Health,
University of Oxford.

Annette McHugh & Pranav Pandya
NHS Fetal Anomaly Screening Programme

A. GENERAL INFORMATION

1. Please identify the ultrasound unit (and trust if applicable) where you currently work and your role:

2. Which identifier best describes your health care setting? (Please check all that apply):

☐ Community Care
☐ Tertiary Care
☐ Private Unit
☐ District General Hospital
☐ University Hospital/Academic Centre

B. QUESTIONS ABOUT THE FIRST TRIMESTER SCAN IN YOUR UNIT

3. In total, how many first trimester ultrasound examinations were performed in your unit over the past one year (between 10^{+0} and 14^{+1})?

4. When does your centre offer routine first trimester anomaly ultrasound screening?

Beginning from: (check one box only)

☐ 10^{+0} weeks GA
☐ 11^{+0} weeks GA
☐ 11^{+2} weeks GA
☐ 12^{+0} weeks GA
☐ 13^{+0} weeks GA
☐ 13^{+6} weeks GA
☐ 14^{+0} weeks GA
☐ 14^{+1} weeks GA
☐ Other:

Offered until: (check one box only)

☐ 10^{+0} weeks GA
☐ 11^{+0} weeks GA
☐ 11^{+2} weeks GA
☐ 12^{+0} weeks GA
☐ 13^{+0} weeks GA
☐ 13^{+6} weeks GA
☐ 14^{+0} weeks GA
☐ 14^{+1} weeks GA
☐ Other:

5. What mode of ultrasound is used routinely for first trimester scans in your unit? (Please check one box only)

☐ Trans-abdominal ultrasound only
☐ Trans-vaginal ultrasound only
☐ Trans-abdominal ultrasound primarily, with use of trans-vaginal probe when required

☐ Trans-vaginal ultrasound primarily, with use of trans-abdominal probe when required

6. How much time is routinely allocated to a first trimester scan for a singleton pregnancy? (Please check one box only)

☐ < 10mins	☐ 10 mins	☐ 15 mins	☐ 20 mins	☐ 25 mins
☐ 30 mins	☐ 35 mins	☐ 40 mins	☐ 45 mins	☐ >45 mins

7. How much time is routinely allocated to a first trimester scan for a multiple pregnancy? (Please check one box only)

☐ < 10mins	☐ 10 mins	☐ 15 mins	☐ 20 mins	☐ 25 mins
☐ 30 mins	☐ 35 mins	☐ 40 mins	☐ 45 mins	☐ >45 mins

8. Apart from ultrasound scanning, does the time allocated to each first trimester scan (as indicated above) include time for any of the following: (Please check all that apply)

☐ Pre-test counselling?
☐ Informed verbal consent?
☐ Post-test counselling/disclosure of findings?
☐ Other:

9. Which of the following assessments are routinely performed as part of your current first trimester fetal ultrasound scanning protocol? (Please check all that apply)

N.B.: Assessments marked with * indicate examinations which are not currently required by NHS FASP first trimester guidance.

☐ Confirmation of fetal viability
☐ CRL measurement for pregnancy dating
☐ Nuchal Translucency
☐ Nasal Bone*
☐ Ductus Venosus Flow*
☐ Tricuspid regurgitation*
☐ Placenta (location)*
☐ Placenta (appearance)*
☐ Head Circumference (HC) measurement (in addition to CRL) *
☐ Bi-parietal Diameter (BPD) measurement (in addition to CRL) *
☐ Evaluation of amnionicity/chorionicity in cases of multiple pregnancy
☐ Visualization of fetal anatomy*

Note: If the 'Visualization of fetal anatomy' box has been checked – please proceed to question 10; If your unit does not assess any fetal anatomy routinely in any women in the

first trimester then please omit questions 10 to 21 and proceed directly to question 22.

10. Assessment of first trimester fetal anatomy in your centre is offered in which of the following cases: (Please check all that apply)

- ☐ Routinely offered to all women
- ☐ For women with previous obstetric history
- ☐ For women with previous family/personal history
- ☐ For women with advanced maternal age
- ☐ For women with maternal risk factors (medication history, T1DM, etc)
- ☐ For pregnancies with raised nuchal translucency? (Eg. NT ⩾ 3.5mm)
- ☐ Any woman deemed to be at higher chance of carrying a fetus with an anomaly
- ☐ Parental request
- ☐ Other:

11. Are women routinely provided with written pre-scan information regarding first trimester screening for fetal anomalies specifically?

- ☐ Yes – women receive a locally developed leaflet about first trimester anomaly screening only
- ☐ Yes – women receive the PHE handout 'Screening tests for you and your baby' only
- ☐ Yes – women receive both the PHE handout 'Screening tests for you and your baby' AND a locally developed leaflet about first trimester anomaly screening.
- ☐ No – women do not receive written pre-scan information prior to first trimester anomaly screening

12. Does your unit provide a formal anatomical protocol for sonographers to use requiring visualization of specific anatomical structures in the first trimester? (Please check one box only)

☐ Yes ☐ No ☐ No pre-set protocol by department – sonographer dependent

13. What anatomical fetal structures are routinely assessed as part of first trimester anomaly screening in your centre? (Please check all that apply)

☐ Head	☐ Face	☐ Neck (additional to NT)
☐ Thorax	☐ Heart	☐ Spine
☐ Stomach	☐ Bladder	☐ Kidneys
☐ Cord Insertion	☐ Limbs	☐ Placenta
☐ Other:		

14. Does your unit advocate routine use of colour flow Doppler for the performance of first trimester anomaly screening?

☐ Yes ☐ No

15. In approximately what % of cases are the objectives of the first trimester anomaly scan unable to be completed (after the first attempt)? (Please check one box only)

☐ <2% ☐ 5% ☐ 10% ☐ 25% ☐ 50% ☐ >50% ☐ Unable to answer

16. Is it policy in your unit to offer a follow-up/repeat ultrasound appointment prior to 18 weeks in cases where all the objectives specific to anomaly screening in the first trimester ultrasound have not been met? (Please check one box only)

☐ Yes ☐ No ☐ Only in specific cases:

17. Are sonographers routinely required to archive/store images relating to visualization of first trimester anatomy (not including NT or CRL)? (Please check one box only)

☐ All images are routinely stored
☐ None of the images are routinely stored
☐ Only images of abnormal or suspicious anatomy are stored
☐ Selected views are stored:
☐ Sonographer dependent
☐ Other:

18. What methods are used for first trimester anomaly image storage? (Please check all that apply)

☐ Thermal image system
☐ Other:
☐ Electronic Digital Archiving System (Eg. PACS)
☐ No images stored

19. Does your unit require sonographers performing first trimester anomaly screening to undergo any form of mandatory training specific to the detection of anomalies prior to 14 weeks GA?

☐ Yes - Formal training provided for sonographers in-house.
☐ Yes - Sonographers required to complete external course. Please indicate which course (eg. FMF, ISUOG, etc.):
☐ No – Formal training specific to first trimester anomaly detection not required for sonographers.
☐ No – Formal training specific to first trimester anomaly detection not required for sonographers BUT they are encouraged to attend external courses to supplement knowledge

and existing skills specific to this area.

20. Is it hospital policy to notify the National Congenital Anomaly and Rare Diseases Registration Service (NCARDRS) regarding anomalies detected in the first trimester?

☐ Yes ☐ No ☐ Only in specific cases:

21. Is there a specific local protocol in place in your unit for management of patients with either a suspected or positively identified fetal anomaly in the first trimester? (Please check all that apply)

☐ Yes – policy advocating immediate disclosure of results after first scan suggesting anomaly.
☐ Yes – policy advocating deferral of disclosure of results to patient until confirmation scan.
☐ Yes – policy of patient referral to second sonographer within unit for confirmation of finding.
☐ Yes – policy of patient referral to GP for further management/investigations.
☐ Yes – policy of patient referral to Obstetrics team for further management/investigations
☐ Yes – policy of patient referral to local FMU for further management/investigations.
☐ No formal policies in place specific to management of first trimester anomalies.

C. ABOUT YOUR SONOGRAPHER TEAM & AVAILABLE RESOURCES:

22. Who performs first trimester ultrasound screening in your unit? (Please check all that apply)

☐ Qualified midwife sonographers
☐ Fetal medicine consultants
☐ Consultant radiologists
☐ Other associate specialists
☐ Qualified radiology sonographers
☐ Fetal medicine fellows
☐ Radiology fellows

23. How many sonographers (radiographer or midwife sonographers) conduct obstetric ultrasound in your unit?

Number: FTE:

24. Of the sonographers included above in Question 23, how many perform first trimester ultrasound?

Number: FTE:

25. How many sonographers in your unit are registered with the Down's Syndrome Quality Assurance Support Service (DQASS)?

26. Do any of your sonographers have Fetal Medicine Foundation (FMF) Certification for the first trimester anomaly ultrasound scan?

- ☐ Yes – All sonographers working in the unit
- ☐ Yes – Some sonographers in the unit - The number of sonographers with FMF certification is (if available):
- ☐ None of the sonographers working in the unit

27. Do sonographers in your unit routinely have access to the following equipment for the purposes of first trimester scanning? (Please check all that apply)

A. High frequency (5-9MHz) trans-abdominal probe (curved transducer)	☐ Yes	☐ No
B. High frequency (5-9MHz) trans-abdominal probe (linear transducer)	☐ Yes	☐ No
C. High frequency (5-12MHz) trans-vaginal probe	☐ Yes	☐ No

28. Regarding the ultrasound machines used in your department for first trimester screening:

A. How many machines are used for first trimester screening?
B. How many of these machines are less than 5 years old?
C. How many of these machines are less than 10 years old?
D. How many of these machines are greater than 10 years old?

29. Does your unit have the capacity and resources to meet current demands for first trimester screening from your catchment area?

- ☐ Yes – we are generally able to meet demand
- ☐ No – we are frequently unable to provide first trimester ultrasound screening during the appropriate gestational age window

30. How often are sonographers in your unit able to undertake DQASS feedback and image review?

- ☐ Always
- ☐ Often
- ☐ Sometimes
- ☐ Rarely

☐ Never

D. ABOUT LOCAL POLICIES IN YOUR UNIT:

31. Does your unit routinely offer women an ultrasound scan prior to 10^{+0} weeks gestational age (GA)? (Please check all that apply)

☐ Yes – offered to all women who have booked prior to 10^{+0} weeks
☐ Yes – offered to women on clinical indication (eg.previous ectopic, chance of multiples, etc.)
☐ Yes – offered to women upon request
☐ No – this is not routine practice in our unit

32. In women who decline screening for Down's syndrome, Edwards' syndrome and Patau's syndrome, is it policy to offer a first trimester ultrasound scan between 10^{+0}-14^{+1} weeks in your unit? (Please select only one answer)

☐ Yes – first trimester ultrasound scan offered between 10^{+0}-14^{+1} weeks
☐ Yes – first trimester ultrasound scan offered – but not necessarily between 10^{+0}-14^{+1} weeks
☐ No first trimester ultrasound scan offered

33. In women who decline screening for Down's syndrome, Edwards' syndrome and Patau's syndrome, is it policy for nuchal translucency to be routinely measured in your unit? (Please select only one answer)

☐ Yes ☐ No

34. In women who decline screening for Down's syndrome, Edwards' syndrome and Patau's syndrome, is there a local policy in place regarding the disclosure of unexpected/incidental ultrasound findings (including enlarged nuchal translucency and/or fetal anomaly findings)? (Please select only one answer)

☐Yes ☐ No

35. Regarding termination of pregnancy (TOP) taking place as a result of findings from first trimester screening undertaken in your unit:

A. Who is the responsible provider?	☐NHS	☐ Independent Unit (eg. BPAS)	☐ Unable to answer	
B. What method of TOP is offered?	☐ Predominantly Medical	☐ Predominantly Surgical	☐ Both medical and surgical options given	☐ Unable to answer
C. What is the setting for TOP?	☐ Predominantly Inpatient	☐ Predominantly Outpatient	☐ Predominantly Day Case	☐Unable to answer
D. Are patients offered option of post-mortem or autopsy after first trimester termination?	☐ Yes	☐ No	☐Unable to answer	

36. Is there anything further you would like us to know about first trimester ultrasound screening in your centre? Any clarifications you'd like to make regarding the answers given above?

Has your unit faced any challenges in implementing first trimester ultrasound screening? Is there anything you feel should be addressed by the UK NSC regarding the evidence for first trimester anomaly ultrasound screening, the NHS FASP regarding the current screening pathway or NHS England as to how first trimester screening is delivered?

Thank you!
