# Supplementary material for: Impact of first-trimester ultrasound on early detection of major fetal anomalies: Nationwide population-based study of over 1 million pregnancies
Source: PLoS Med. 2025 Nov 25;22(11):e1004709. doi: 10.1371/journal.pmed.1004709 (PMC12646414; doi:10.1371/journal.pmed.1004709)
Supplement: S2 Appendix — (PDF) [file pmed.1004709.s004.pdf]

Appendix 2 – ICD-10 codes used to identify fetuses within NCARDRS database affected by one of 14 congenital anomalies of interest in this study.

Condition	Associated ICD10 codes	Notes/limitations
Acrania	Q00* Anencephaly and similar malformations	Includes iniencephaly in line with EUROCAT<br>Any cases with anencephaly as part of TRAP sequence are excluded
Spina bifida	Q05* Spina bifida	All spina bifida including open, closed and where not specified
Encephalocele	Q01* Encephalocele	
Facial Clefts	Q36* Cleft lip<br>Q37* Cleft palate with cleft lip	
Diaphragmatic hernia	Q79.0* Congenital diaphragmatic hernia	
Gastroschisis	Q79.3 Gastroschisis	
Exomphalos	Q79.2 Exomphalos	
Transposition of Great Arteries	Q20.3 Transposition of the great arteries, TGA	
Atrioventricular Septal Defects	Q21.2* Atrioventricular septal defect, AVSD balanced and unbalanced	
Tetralogy of Fallot	Q21.3 Tetralogy of Fallot, TOF<br>Q21.82 Pentalogy of Fallot (or TOF with ASD)	
Hypoplastic Left Heart Syndrome	Q23.4 Hypoplastic left heart syndrome, HLH	
Lethal Skeletal Dysplasias	Q77.0 Achondrogenesis, type I and type II<br>Q77.1 Thanatophoric short stature<br>Q77.2* Short rib syndrome<br>Asphyxiating thoracic dysplasia [Jeune]<br>Jeune's syndrome<br>Q77.8 Other osteochondrodysplasia with defects of growth of tubular bones and spine<br>Acrodysostosis<br>Kniest dysplasia<br>Metatropic dwarfism	Codes taken from Boyd et al, 2011<br>Excluding mild osteogenesis imperfecta

	Metaphyseal chondrodysplasia<br>Q78.0 Osteogenesis imperfecta	
Bilateral renal agenesis	Q60.1 Renal agenesis, bilateral	Excluding post-screening atrophy
Limb reduction	Q71 Reduction defects of upper limb<br>Q72 Reduction defects of lower limb<br>Q73 Reduction defects of unspecified limb	

References:

Boyd PA, Tonks AM, Rankin J, Rounding C, Wellesley D, Draper ES, BINOCAR Working Group. Monitoring the prenatal detection of structural fetal congenital anomalies in England and Wales: register-based study. Journal of medical screening. 2011 Mar;18(1):2-7.
