# Supplementary material for: Impact of first-trimester ultrasound on early detection of major fetal anomalies: Nationwide population-based study of over 1 million pregnancies
Source: PLoS Med. 2025 Nov 25;22(11):e1004709. doi: 10.1371/journal.pmed.1004709 (PMC12646414; doi:10.1371/journal.pmed.1004709)
Supplement: S3 Appendix — (PDF) [file pmed.1004709.s005.pdf]

Appendix 3 - Screening and protocol use in each geographical region.

Table indicating which anatomical structures are formally assessed in the first trimester by those hospital trusts using a protocol in each region. Each region is represented anonymously represented by the letter A to I.*For one unit in Region D, responses indicated that early anomaly screening was routinely offered with a formal anatomical protocol in use, however this protocol was not made available and therefore could not be assessed. The nine available protocols for Region D were therefore included in the analysis. **For one unit in Region I, responses indicate that early anomaly screening was routinely offered without a formal protocol in use and this was not made available for analysis. The three available protocols for Region I were therefore included in the analysis. ^Of the 83 units included in this analysis, only 81 protocols were available for analysis.

	Health Region									
	A	B	C	D	E	F	G	H	I	All
**Total number of responding hospital trusts in each region (n)**	16	17	16	4	11	14	11	8	13	110
**Trusts offering early anatomical assessment (n, %)**	16 (100)	17 (100)	13 (81)	3 (75)	8 (73)	10* (71)	6** (55)	4 (50)	6 (44)	83^ (75)
**Trusts using a formal anatomical screening policy/ protocol (n, %)**	14 (88)	10 (59)	8 (62)	2 (66)	5 (45)	6 (60)	2 (33)	3 (75)	2 (33)	52 (63)
**Protocol includes examination of:**										
Head	16 (100)	17 (100)	13 (100)	2 (66)	8 (100)	9 (100)	5 (100)	4 (100)	6 (100)	80 (99)
Limbs	15 (94)	17 (100)	13 (100)	3 (100)	7 (88)	9 (100)	5 (100)	3 (75)	5 (83)	77 (95)
Cord Insertion	16 (100)	14 (82)	13 (100)	3 (100)	6 (75)	9 (100)	5 (100)	4 (100)	4 (66)	74 (91)
Stomach	16 (100)	17 (100)	9 (69)	2 (66)	3 (38)	6 (66)	0 (0)	2 (50)	3 (50)	58 (72)
Bladder	15 (94)	16 (94)	6 (46)	3 (100)	3 (38)	5 (56)	0 (0)	2 (50)	3 (50)	53 (65)
Heart	4 (25)	6 (35)	1 (8)	0 (0)	0 (0)	1 (11)	0 (0)	1 (25)	1 (17)	14 (17)
Spine	4 (25)	6 (35)	0 (0)	0 (0)	0 (0)	1 (11)	0 (0)	1 (25)	1 (17)	13 (16)
